# The Fallacy of Chasing after Work-Life Balance

**DOI:** 10.3389/fped.2014.00026

**Published:** 2014-03-31

**Authors:** Andreas Schwingshackl

**Affiliations:** ^1^Division of Critical Care Medicine, Department of Pediatrics, University of Tennessee Health Science Center, Memphis, TN, USA

**Keywords:** work, life, balance, work-life balance, pediatrics, critical care, burn out

“There is no such thing as work-life balance. Everything worth fighting for unbalances your life.”

— Alain de Botton

As a pediatric intensivist and clinician-scientist, finding a balance between work and life is essential to my professional success and my personal happiness. Failure to achieve a healthy balance will result in burn-out. So I was warned all these years since the first day of medical school. In 2013, 180 articles documented that physicians are highly dissatisfied with their jobs. This is a testament to how poorly we are balancing work and life (1–4). Interestingly, to my best knowledge, to date only 12 articles have been published specifically addressing work-life balance in Pediatrics (5–16).

Since entering medical school over two decades ago, I reached the conclusion that the concept of work-life balance acts as quicksand in our professional and personal lives resulting in slow drowning in frustration, depression, and exhaustion. The harder we fight the deeper and quicker we sink. Why are we taught to strive for work-life balance in the first place? The entire future of our modern “24/7” society appears to revolve around mastering this concept, with the ultimate promise that – once achieved – we will all be compensated for the misery and sacrifices that we endured along the way. Astoundingly, a recent study reported an almost 20% higher job dissatisfaction rate for physicians than for the general US population (17).

In reality, the concept of work-life balance is imposed upon us by corporations, companies, and employers with the primary intent to maximize our productivity margins at the work place, *not* to improve our emotional or physical well-being. The cold truth is that all corporations are specifically designed to maximize their financial profit, not the happiness or well-being of their employes. For this vitally important reason, we can under no circumstances leave it to our employers to determine the quality of our own lives (18). To maintain intellectual autonomy as individuals, the task of creating our own happiness has to remain in our own hands. Some employers offer a free smartphone, a tabloid, free daycare, or other after-hour programs as part of their “benefits” package, but in reality they just provided us with the means to spend even more time at work or doing work for them.

The new buzz-word in Medicine is “flextime” (19, 20). Besides the fact that for some medical specialties, such as Intensive Care, flextime is a less viable concept than for other specialties, the fact still remains that if *my* time is flexible *somebody* has to work the hours that I find inconvenient. This rarely results in two happy employes. Residents and fellows are now restricted to 80 work hours per week (21) and after subtracting even as little as 6 h of sleep per night, they are left with 46 h per week to fill with “life.” Therefore, medical trainees have at best half as many hours available for leisure as they are required to spend at work. Clearly, achieving a work-life balance under these conditions is extremely unlikely, probably even impossible. Importantly, despite work hour restrictions physicians’ dissatisfaction with both their jobs and lives is actually at an all-time high and rising (22, 23). One can argue that residency and fellowship are only temporary occupations but in reality I do not remember working any less in medical school or now as an attending. The increased amount of responsibilities assigned to a physician once out-of-training actually accentuates rather than diminishes our daily stress level and results in further spillover of work-time into leisure-time.

As we follow our peers’ instructions trying to balance work and life, the question arises: *when* should we achieve this balance and *How do we know* we achieved it? Since most of us enter the workforce as teenagers, would our high school or medical school years be a good time to start embracing this concept? Our early professional career years? At mid-career level? After retirement? While common sense tells us that the pursuit of work-life balance should occur during *all* stages of our careers, we all have a tendency to constantly postpone any significant improvement in our daily quality of life until after the current project is completed, after this service week, after the next promotion, after the kids are out of the house. Just how poorly we balance our work and life throughout our careers was highlighted in a recent study showing that early career physicians had the lowest satisfaction rate with their overall career choice, and mid-career physicians reported the lowest satisfaction rate with their specialty choice and their work-life balance (24). Alarmingly, similar job dissatisfaction rates have been reported for medical students and interns (25). Particularly, the high institutional demands for the new generation of clinician-scientists, which are deeply rooted in our professional culture, add further barriers to a healthy work-life balance (26). Clinician-scientists face additional challenges dealing not only with the separation between work and life but also between clinical care and research. A key reason for early career physicians to leave academic medicine is in fact the disconnection between their own priorities and those of the dominant culture of academic medicine (27). It is obvious that as physicians we are facing tremendous struggles throughout *all* career stages in implementing what would seem the natural number one priority for our health and happiness, a balanced life-style.

Personally, I concluded that the never-ending chase after work-life balance actually accumulates much more frustration than satisfaction. Regardless of this depressing conclusion, we may not at all be doomed if the true payoff reveals itself not in the chase but in the achievement of work-life balance. This leads me to the second question: *how would we know* that we achieved it? Exactly how much life do we need to balance our work? Will we just wake up one day and feel “balanced”? Unfortunately, the absence of an objective outcome measure makes the chances of ever achieving this goal rather elusive. As the movers and shakers of our industrialized nations continue to promote the work-life balance concept as the Holy Grail of the twenty-first century go-getter mentality, engraining into our minds that a more balanced and happier life lies just around the corner, I cannot help but getting reminded of the entrance gate at Auschwitz displaying “Arbeit Macht Frei” (Work Will Liberate You”). The pursuit of a concept that is intangible and lacks validation is unlikely to result in anything but a sense of failure and helplessness, or what most of us know as “burn-out”.

Twenty-four years after entering medical school, after uncountable reminders by my peers at each step of my career that *this right now* is actually the best time of my life, lecture after lecture from medical school through residency and fellowship all the way to today’s Faculty Development Program in my Department, I am reminded that without finding that magical work-life balance I cannot, and will not, succeed in this stressful and demanding profession (28, 29). Instead, over the past two decades, I realized that regardless of the number of programs our society develops to promote the concept of a healthy work-life balance it is an indisputable fact that certain career pathways, including modern academic medicine, are inherently incompatible with spending the majority of our time with our families and children, or engaging in certain hobbies such as traveling the world.

All that being said, over the past few years I have adapted a new approach to a healthier and happier life-style that resulted in much greater job satisfaction. While the medical education system has never offered me an alternative solution to living a fulfilled life except the pursuit of work-life balance, I have come to the conclusion that as long as there is a polarity in our daily lives between what we consider work and what we consider life there will always be conflict. Only the abolition of this dichotomy will establish harmony in our lives. Once I was able to integrate rather than separate all my daily activities, harmonize rather than divide my time not only between work and life but also between clinical care and research, the pursuit of balance shifted from work-life to life-nature-universe. The result was an overwhelming daily feeling of “balance.” Buddenberg-Fischer recognized in 2008 that “*a well-balanced integration (not separation) of professional and private life is an essential goal for the new generation of doctors*” (30).

The constant pursuit of work-life balance actually worsens rather than improves our quality of life by adding additional, often unrealistic, expectations to our already stressful lives. Uncountable websites and publications promote quick fixes for the “unbalanced” health care worker (31–33). The question remains: can a successful clinician-scientist really eat six small meals a day? Work-out four times a week? Attend all family functions? Spend regular quality time with friends? The root of the problem lies in the fundamental assumption that life is good and work is bad, which is the main reason why we need a work-life balance in the first place. This distinction also implies that life only occurs whenever we are not at work, demoting the importance of work in our lives and projecting unrealistic expectations onto our time-off-work. The feeling that work is externally imposed onto us causes resentment against this activity and victimizes us as employes implying that we are forced to work against our free will. It is a fact that we spend more time at work than with our partners, our families, or in bed. Therefore, to label the majority of our time as unwanted and burdensome translates into increasing exhaustion and frustration at the workplace. This creates enormous pressure on our leisure-time to compensate for all the negative energy that accumulates at work. In return, the inability to accomplish all the regenerating goals, we had set for today results in further desperation and inevitable failure.

All humans have an intrinsic desire to create, to build, and to leave a mark and an impression, as a matter of fact to work. As humans, we have always searched the contact with other humans. We have a desire to share our experiences, creations, and achievements with others. If we consider our time at work as just that, as time with colleagues and friends that allows us to create, to build, and to leave a mark and an impression, then suddenly life has taken over work, and the pursuit of a work-life balance becomes an obsolete concept. Our happiness may in fact have nothing to do with finding balance but much rather, as John Irving writes, with *finding a way of life we love and having the courage to live it*.

If as physicians we stand behind the personal statements we wrote as medical students and our eloquent speeches during job interviews describing that special day we realized we were destined to become a doctor and care for sick children, then we all clearly chose this profession out of free will. If that premise holds true, then the hours spent treating sick children are part of our lives just as much as the hours sipping on a glass of wine, going on a family vacation, or fishing with our buddies. Suddenly, the border between life and work vanished, work became life, and life became work. We all have some better and some worse days, but as physicians by the end of each day we will have made a difference in at least one child’s life. If at that moment, we pause for a second acknowledging this incredible achievement, recognizing that we made this world a better place for somebody today, we will experience the indescribable privilege of feeling balanced every day.

Two children died last night during my call in the ICU. One family saved the lives of four children by donating their son’s organs. The other family told my team what an honor it was for them to meet us and they will never forget how we helped them cope with the tragic death of their 12-year-old daughter. I am tired, exhausted, and hungry. But right now, I am balanced.
